# Delayed dorsal scapular artery hematoma following blunt thoracic trauma: a case report and review of the literature

**DOI:** 10.1186/s13256-022-03400-z

**Published:** 2022-05-04

**Authors:** Anna Maria Florescu, Ane Brøndum Lange, Erik Gudmann Steuble Brandt, Anders Vestergaard Krusenstjerna-Hafstrøm, Henrik Vad, Peter Hallas

**Affiliations:** 1 Department of Neurology, Rigshospitalet Glostrup, Copenhagen University Hospital, Copenhagen, Denmark; 2 Department of Internal Medicine, Sygehus Sønderjylland, Sønderborg, Denmark; 3 Department of Diagnostic Radiology, Holbæk Hospital, Holbæk, Denmark; 4 Department of Emergency Medicine, Holbæk Hospital, Holbæk, Denmark; 5 Department of Cardiothoracic Surgery, Rigshospitalet, Copenhagen University Hospital, Copenhagen, Denmark; 6 Department of Neurology, Rigshospitalet Glostrup, Faculty of Health and Medical Sciences, University of Copenhagen, Valdemar Hansen Vej 5, 2600 Glostrup, Denmark

**Keywords:** Blunt chest trauma, Dorsal scapular artery, CT angiography, Delayed complication

## Abstract

**Background:**

The present case contributes to the limited literature on delayed chest wall hematomas following blunt trauma. The literature review provides a summary of similar previously reported cases.

**Case presentation:**

We report the case of a 59-year-old Caucasian male who presented to the emergency department with a rapidly expanding chest wall hematoma. Six weeks earlier, he had sustained multiple rib fractures and a pneumothorax after falling 4 m from a ladder. Computed tomography angiography was used to identify two sources of active bleeding on the left dorsal scapular artery. The patient underwent surgery with evacuation of the hematoma and ligation of the artery. The patient was hospitalized for 3 days and recovered with no sequelae.

**Conclusions:**

A literature review revealed eight previously reported cases of chest wall hematomas exterior to the endothoracic fascia following blunt trauma. Most cases were initially diagnosed by computed tomography of the chest and finally by angiogram. Management options range from surgical drainage to angiographic embolization. This case is unusual regarding the delay in the development of the hematoma and illustrates the importance of considering this diagnosis even weeks after relevant trauma.

## Background

Blunt injuries account for the majority of chest traumas and cause a range of complications, including rib fractures, pneumothorax, hemothorax, and chest wall hematomas [1]. Arterial bleeding commonly presents acutely within minutes or hours after injury, whereas delayed bleeding, defined as onset more than 24 hours after injury, is a known but rare occurrence [2, 3]. This case report’s main objective is to highlight the importance of considering chest wall hematomas as a delayed complication to blunt trauma.

## Case presentation

A 59-year-old Caucasian male was admitted to the emergency department with a rapidly expanding mass in the thoracic wall in relation to the inferior part of the left shoulder blade. His medical history included mild renal insufficiency and epilepsy. He did not take any antiplatelet or anticoagulant medication. Six weeks earlier he fell 4 m from a ladder, resulting in multiple rib fractures and a left-sided pneumothorax diagnosed on chest radiography. The pneumothorax was treated with a chest drain placed in the left anterior axillary line at the level of the papilla. Pain-relieving medication included paracetamol, ibuprofen, and oxycodone, and the patient was released from the hospital after 5 days of treatment.

The mass in the thoracic wall had appeared the previous evening about 8 hours before the patient came to the hospital and kept expanding slowly during his stay in the emergency department. He had pain in his left shoulder, and his left-hand fingers felt cold and tingling with no further neurological symptoms. A clinical examination revealed a skin-colored, sore swelling approximately 10 cm in diameter in relation to the inferior angle of his left shoulder blade. Vital parameters were normal. Blood results showed hemoglobin levels of 9.18 g/dL compared with 11.92 g/dL 6 weeks earlier.

Thoracic computed tomography angiography (CTA) revealed a large hematoma in relation to the left scapula located between the costae/intercostal muscles and the serratus anterior muscle. The hematoma measured 7 cm × 14 cm × 18 cm (anteroposteriorly × right–left × craniocaudally) equaling a volume of approximately 930 mL (Eq. 1). Two sites of active bleeding were identified on the left dorsal scapular artery located between the medial border of the scapula and numerous rib fractures. The CTA showed rib fractures from the 3rd to the 12th left rib, including double rib fractures of the 4th to 7th rib, corresponding to the shape of the scapula (Paraclinical photos).

The patient remained stable and was transferred to the department of cardiothoracic surgery. Open exploration was chosen because of compression symptoms. At primary surgery, the hematoma was evacuated beneath the left latissimus dorsi. The bleeding source was not found, and the resulting cavity was packed with gauze binding. At second look the following day, the scapula was lifted, coagulated blood was removed, and the bleeding sources on the left dorsal scapular artery were identified and ligated. The patient was discharged from the hospital after a total of 3 days. Six months later, the patient was well with no sequelae.

### Aims of the systematic literature review

We conducted a review of the literature to identify how previous cases of chest wall hematomas exterior to the endothoracic fascia following blunt trauma have been diagnosed and managed. We hypothesized that most cases were diagnosed by CTA of the chest and managed by surgical drainage similarly to our case.

## Methods

### Search criteria and study selection

The literature search was conducted in PubMed to identify eligible articles. The search was divided into four main categories using MeSH term searches. The four categories were (1) “Thorax,” (2) “Blunt trauma,” (3) “Hemorrhage,” and (4) “Arterial bleeding.” MeSH terms within one of the four categories were combined using Boolean operators “OR.” Finally, all four categories were combined using the Boolean operator “AND.” Two authors (AMF, ABL) conducted the search individually to secure validation of the method. The MeSH term search was followed by an “All Fields” search in PubMed to expand the search. Lastly our own reference list was screened.

*MeSH search string* (“Scapula”[MeSH Terms] OR “Thorax”[MeSH Terms] OR “Intercostal Muscles”[MeSH Terms] OR “Thoracic Injuries”[MeSH Terms]) AND (“wounds, nonpenetrating”[MeSH Terms] OR “Wounds and Injuries”[MeSH Terms] OR “Accidental Falls”[MeSH Terms]) AND (“Blood”[MeSH Terms] OR “Hemorrhage”[MeSH Terms] OR “Hematoma”[MeSH Terms]) AND (“Thoracic Arteries”[MeSH Terms] OR “Computed Tomography Angiography”[MeSH Terms] OR “Aneurysm”[MeSH Terms])

*All fields search string* (scapular artery) AND (blunt trauma)

### Inclusion and exclusion criteria

Inclusion criteria: (a) articles in English, (b) articles published after year 2001 (to validate common use of CTA), (c) hematomas exterior to the endothoracic fascia, (d) mechanism: blunt trauma, (e) confirmed diagnosis. Exclusion criteria: (a) hemothorax, (b) cardiac tamponade, (c) unknown vessel, (d) concomitant hematomas interior to the endothoracic fascia, (e) concomitant penetrating trauma, (f) cases in children (< 18 years of age).

**Fig. 1:**
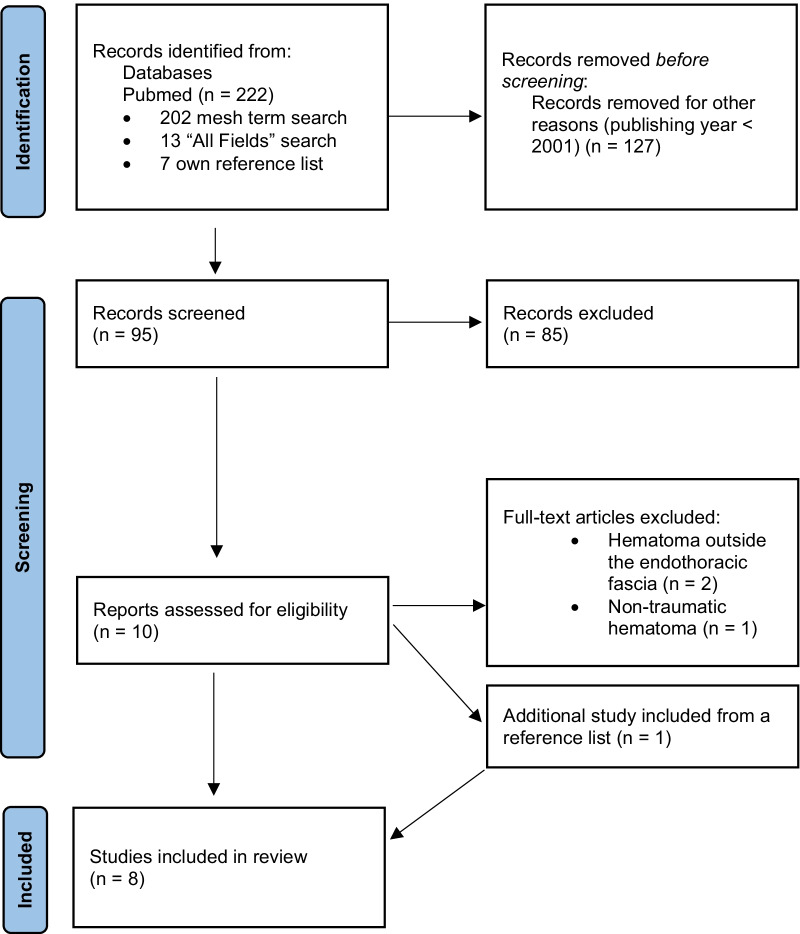
PRISMA flow chart

All papers retrieved from the database were screened by title and abstract by two authors (AMF, ABL). Then, full texts of relevant articles were screened by two authors (AMF, ABL) to identify the papers that satisfied the eligibility criteria for the review. The identified publications references were hand-checked to find additional studies. Studies were then excluded or kept on the basis of their consistency with the inclusion and exclusion criteria. The PRISMA flow diagram was used to record the selection process (Fig. 1).

Information was retrieved on mode of diagnosis, which artery was damaged, whether it was in relation to any fractures, and how the hematoma was managed (Table 1). Information about treatment with anticoagulant therapy was also retrieved since anticoagulants increase the likelihood of bleeding.

**Table 1:** Diagnosis and management of chest wall hematomas exterior to the endothoracic fascia following blunt trauma

Author, year	Study design	Number of patients	Mechanism of injury	Blood thinners	Primary tool of diagnosis	Final tool of diagnosis	Blood vessel	Hematoma in relation to	Treatment
Jin *et al*., 2021 [9]	Case report	1	Motorized scooter crash 2 weeks earlier	No	Chest CT	Angiogram	A pseudoaneurysm on the left dorsal scapular artery	Displaced left third rib fracture	Angiographic embolization
Gorospe *et al*., 2016 [10]	Case report	1	Fall 2 months earlier (unknown height)	No	Chest CT	Angiogram	A pseudoaneurysm on a branch of the right dorsal scapular artery	Multiple nondisplaced right rib fractures, unknown location	Angiographic embolization
Sato *et al*., 2016 [5]	Case report	1	Motor vehicle accident 22 days earlier	No	Chest CT	Angiogram	Rupture of a branch of the left suprascapular artery	Posterior fractures of the left 4th, 5th, 6th, 7th, and 8th ribs	Angiographic embolization
Yu *et al*., 2015 [11]	Case report	1	Fall onto the handlebar during bicycle riding same day	Aspirin	Chest CT	Angiogram	A pseudoaneurysm on a branch of the right anterior humeral circumflex artery	No report	Angiographic embolization with subsequent surgical drainage
Prater *et al*., 2010 [12]	Case report	1	Automobile accident 2 years earlier	Unknown	Chest CT	Angiogram	A pseudoaneurysm on the suprascapular artery	Left clavicle fracture	Angiographic embolization
Patel *et al*., 2009 [13]	Case report	1	High-speed front–end motor vehicular collision same day	Unknown	Chest CT	Angiogram	A pseudoaneurysm on a perforating branch of the left internal mammary artery	No report	Angiographic embolization
Suh *et al*., 2009 [14]	Case report	1	Pounding her own chest same day	No	Contrast-enhanced chest CT	Contrast-enhanced chest CT	Rupture of a perforating branch of the left internal mammary artery	No report	Hematoma removed through a limited subaxillary incision
Antevil *et al*., 2006 [15]	Case report	1	Motor vehicle crash same day	Unknown	Contrast-enhanced chest CT	Angiogram	Rupture of an accessory scapular branch of the right axillary artery and the right lateral thoracic artery	Multiple right-sided rib fractures	Angiographic embolization

## Results

As seen from the PRISMA flow chart (Fig. 1), the search yielded 222 articles (30 June 2021). Ninety-five of these were selected after titles and abstracts were screened. In the secondary screening, ten papers were reviewed in full length, and three of these were excluded for specific reasons, detailed in Fig. 1. One additional paper was included by checking references. In total, eight case reports involving eight patients were included.

The results are presented in Table 1. As seen from Table 1, five out of eight previous cases of chest wall hematomas exterior to the endothoracic fascia were due to pseudoaneurysms following blunt trauma, whereas three were due to vessel rupture, likely caused by nearby fractured ribs. Pseudoaneurysms were due to trauma on the same day and up to 2 years later. Two of the cases with vessel rupture were due to immediate rupture and one 22 days after relevant trauma.

All cases were initially diagnosed with a chest computed tomography (CT), two of those with a contrast-enhanced scan. Seven out of eight cases were finally diagnosed with an angiogram, and the last with a contrast-enchanted chest CT. Seven out of eight cases were treated with angiographic embolization of the artery; one of these underwent subsequent surgical drainage. The last case was removed surgically through a limited subaxillary incision.

## Discussion

This case illustrates a common complication following blunt thoracic trauma in terms of a chest wall hematoma [1] but with an uncommon presentation in terms of the time delay counting 6 weeks from initial trauma to the development of the hematoma. As illustrated in the literature search, it is important to consider this diagnosis even years after initial trauma and treat it adequately to reduce disability or death. At initial hospital discharge, patients suffering blunt thoracic trauma should be informed about potential delayed complications and the importance of seeking medical advice promptly.

There are two possible explanations for the time delay in this case: initial trauma caused two pseudoaneurysms on the dorsal scapular artery [4]; alternatively, a nearby fractured rib disrupted the vessel on the day of the second hospitalization [5]. The latter is a plausible explanation owing to the location of the hematoma in relation to the fractured ribs. A computed tomography (CT) with contrast of the chest could have identified the pseudoaneurysms at initial hospitalization, and in that case the patient could have avoided a second hospitalization.

A CT scan is a sensitive diagnostic tool for hemothorax, pneumothorax, pulmonary contusions, and chest wall hematomas as well as identifying sources of bleeding [6]. This case illustrates how a low-dose contrast chest CT scan could be considered as a primary imagining tool in patients with suspected severe thoracic trauma such as a fall from heights. The decision to use an initial CT scan over chest radiography should always be made thoughtfully, considering benefits such as diagnosing occult life-threatening injuries as well as the disadvantages in terms of higher costs and radiation burden and the risk of overdiagnosis and overtreatment [7]. Most importantly CT chest should be the first choice if patients present with delayed complications after rib fractures, since chest radiography may miss a chest wall hematoma [5].

In terms of treating a chest wall hematoma, angiographic embolization is an effective and minimally invasive procedure and is commonly performed to stop bleeding from a variety of vessels [5]. However, owing to compression symptoms, our patient needed surgical drainage of the hematoma in addition to a ligation of the artery.

## Conclusion

Delayed hematoma from a chest wall artery is a rare complication to blunt thoracic trauma. We present a case where a hematoma of approximately 0.93 L accumulated rapidly 6 weeks after initial trauma due to left dorsal scapular artery bleeding. A review of the literature revealed eight similar cases, five of which were due to pseudoaneurysms and three due to vessel rupture. A contrast chest CT is a useful diagnostic tool for chest wall hematomas, whereas CTA can be used to diagnose hematomas as well as the bleeding sources. The patients should be treated either at a department of cardiothoracic surgery with open exploration or by an interventional radiologist with endovascular embolization [8].

In conclusion, this case report contributes to the relatively limited literature on delayed chest wall hematomas and highlights the importance of considering this diagnosis even weeks after relevant trauma. The systematic literature search provides an overview of diagnostic and management options of chest wall hematomas exterior to the endothoracic fascia.

### Equation 1

The blood volume was calculated as an ellipsoid: $\frac{4}{3} \times\uppi \times \mathrm{radius }1 \times \mathrm{radius }2 \times \mathrm{radius }3.$ In this case: $4.2 \times 3.5\times 7\times 9=926.1\mathrm{ mL}.$ This is equal to the volume of a cube × 0.53.

### Paraclinical photos

The dorsal scapular artery supplies the levator scapulae and rhomboid muscles and is part of the scapular arterial anastomosis. Most frequently, it branches from the second or third part of the subclavian artery, rarer from the transverse cervical artery.

*Thoracic imaging.* **A** A 3D reconstruction of the CTA shows two spots of active arterial bleeding (green arrow) and multiple rib fractures. **B** A 3D reconstruction of the CTA shows spots of active arterial bleeding (red arrow) within the large subscapular hematoma. In addition, multiple rib fractures are shown. **C** A maximum-intensity projection (MIP) reconstruction of the CTA shows two spots of active arterial extravasation of contrast medium (active bleeding). **D** and **E** The CTA shows two focal spots of active arterial bleeding (contrast medium extravasation). **F** A radial rib range reconstruction of the CTA shows rib fractures from the 3rd to the 12th left rib and several right-sided rib fractures.

**Figure:**
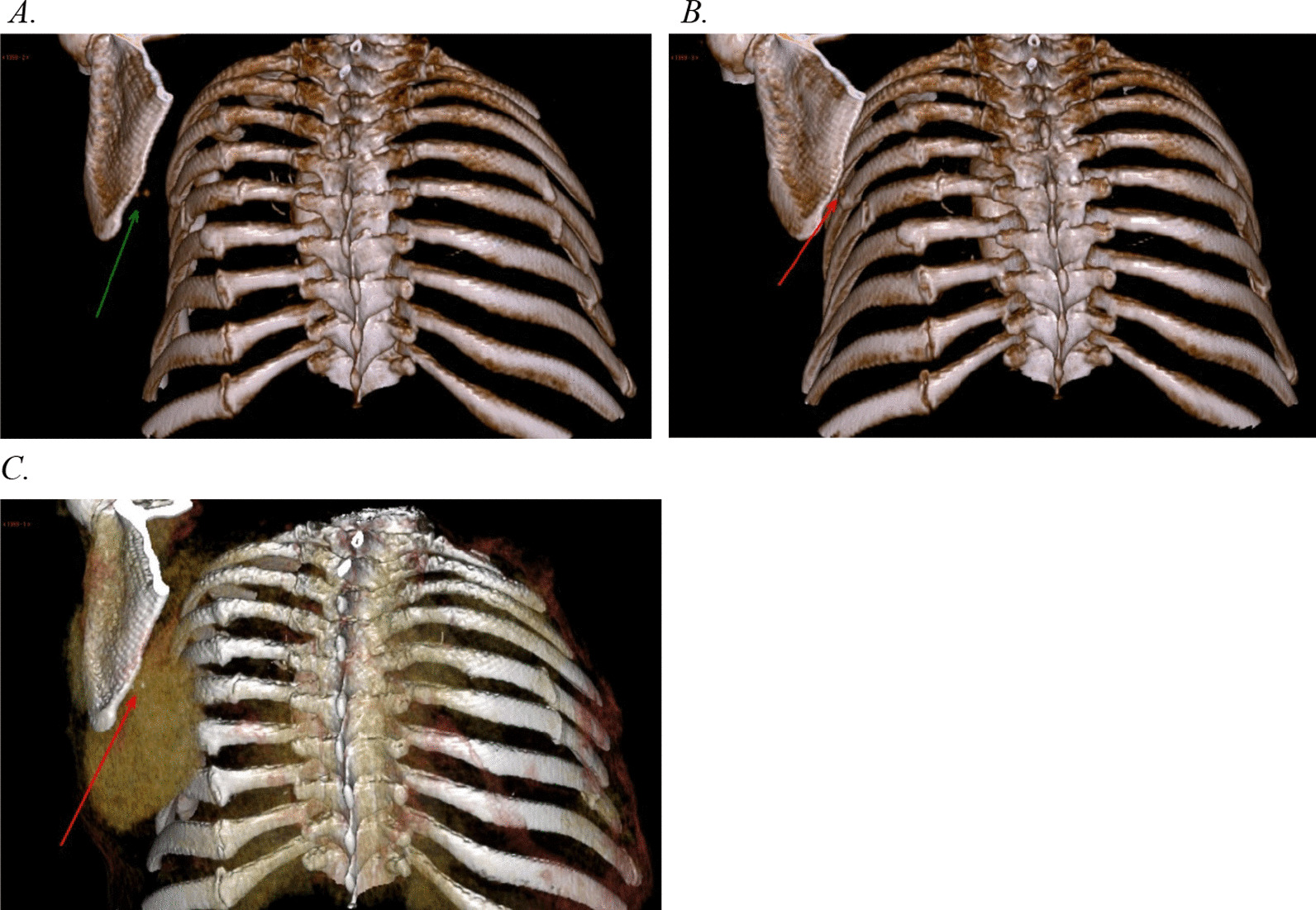

**Figure:**
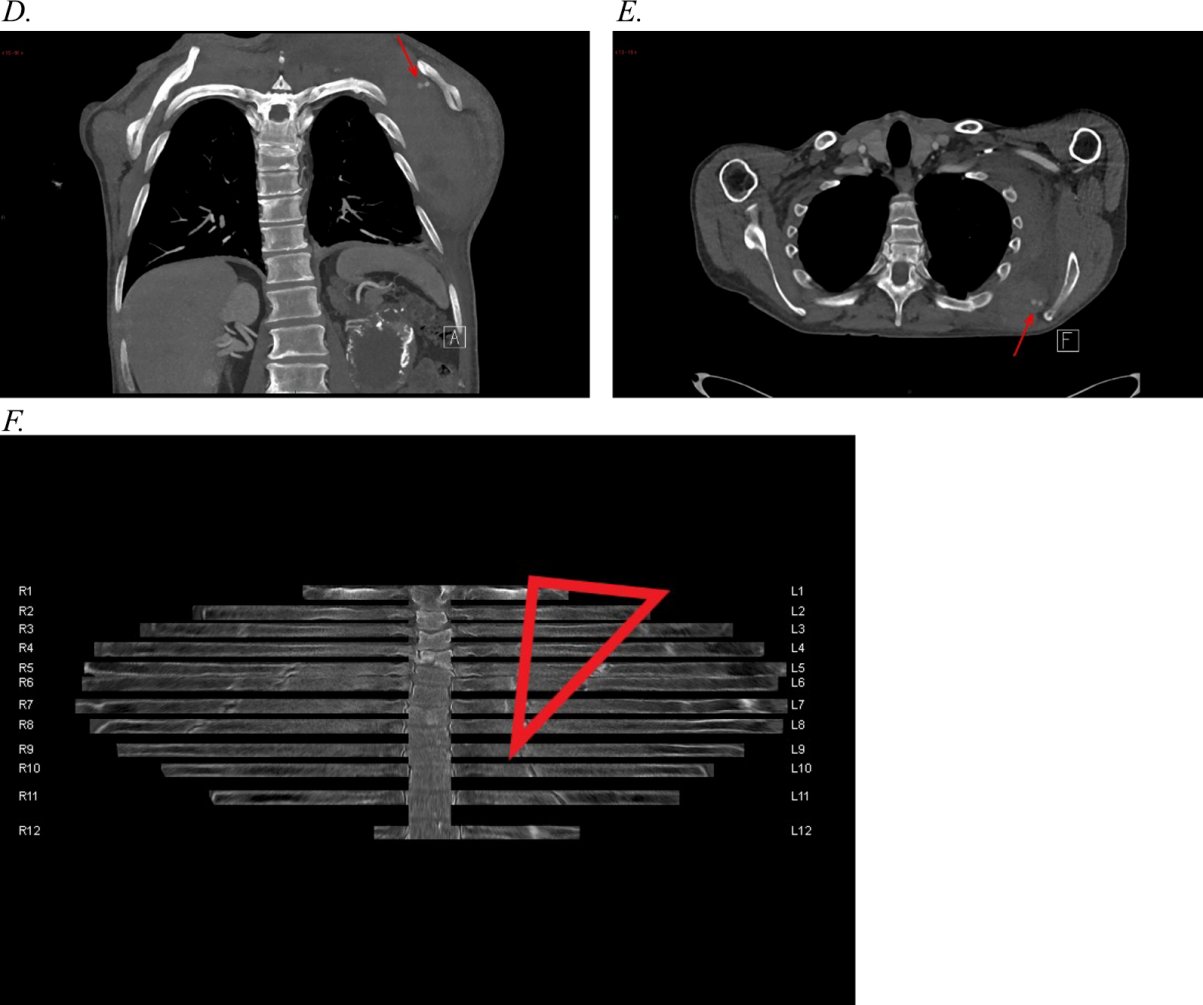

## Data Availability

Not applicable.

## Abbreviations

CTA: Computed tomography angiography
CT: Computed tomography
MIP: Maximum-intensity projection
